# High-quality genome assembly of *Metaphire vulgaris*

**DOI:** 10.7717/peerj.10313

**Published:** 2020-11-12

**Authors:** Feng Jin, Zhaoli Zhou, Qi Guo, Zhenwen Liang, Ruoyu Yang, Jibao Jiang, Yanlin He, Qi Zhao, Qiang Zhao

**Affiliations:** 1 College of Rehabilitation Sciences, Shanghai University of Medicine and Health Sciences, Shanghai, China; 2 Shanghai Key Laboratory of Molecular Imaging, Shanghai University of Medicine and Health Sciences, Shanghai, China; 3 Department of Agriculture and Biology, Shanghai Jiao Tong University, Shanghai, China

**Keywords:** Genome assembly, *Metaphire vulgaris*, Whole genome sequencing, Hi-C, Genome duplication, Hox, Lumbrokinase

## Abstract

Earthworms enrich the soil and protect the health of their ecological environment. Previous studies on these invertebrates determined their protein content, hormone secretions, medicinal value, and ecological habits, but their whole genomic sequence remains incomplete. We performed whole genome sequencing of *Metaphire vulgaris* (Chen, 1930), which belongs to the genus *Metaphire* of the family Megascolecidae. The genome assembly was 729 Mb, with a N50 contig size of 4.2 Mb. In total, 559 contigs were anchored to 41 chromosomes according to the results of Hi-C (High-throughput Chromosome Conformation Capture) technology, which was confirmed by karyological analysis. A comparison of the genomic sequences and genes indicated that there was a whole-genome duplication in *M. vulgaris* followed by several chromosome fusion events. Hox genes and lumbrokinase genes were identified as partial clusters surrounding the genome. Our high-quality genome assembly of *M. vulgaris* will provide valuable information for gene function and evolutionary studies in earthworms.

## Introduction

Earthworms are terrestrial invertebrates belonging to Oligochaeta in the phylum Annelida. There are more than 3,000 species of earthworm in the world, with more than 600 species found in China alone (Csuzdi, 2012; Jiang & Qiu, 2018). Earthworms burrow in the soil, decompose organic matter, and create ideal conditions for the growth and reproduction of soil microorganisms. They are particularly important for soil enrichment and protecting the health of their surrounding ecological environment. Many countries use earthworms to process domestic and organic wastes, and purify sewage. Earthworms are also used in traditional Chinese medicine (TCM) to treat a variety of diseases, and can be used as a high-protein feed.

A number of earthworm studies have focused on their protein content, hormone secretions, medicinal value, and ecological habits but only a few studies have investigated earthworm genomics. Zwarycz et al. (2015) performed whole genome sequencing on *Eisenia fetida* (Savigny, 1826), an earthworm from the family Lumbricidae. And the annotation study were conducted by Paul et al. (2018), although the quality of the assembled genome sequences was not of a sufficient quality for downstream analyses (N50 = 1.85 Kb). Bhambri et al. (2018) sequenced a different strain of *E. fetida* and conducted genome-wide analyses and transcriptome studies. This study also failed to produce a genome assembly sufficient for accurate gene annotation (N50 = 9.31 Kb).

*Metaphire vulgaris* (Chen, 1930) is called ”Hu dilong” in Traditional Chinese medicine (Chinese Pharmacopoeia Commission, 2015) and belongs to the genus *Metaphire* of the family Megascolecidae. It is commonly found in many Chinese provinces, including in Jiangsu, Shanghai, Zhejiang, and Guizhou. It is 120–215 mm long and 5–8 mm wide with body segments 90–124 mm in length. We conducted whole genome sequencing of *Metaphire vulgaris* by combining the single-molecule long sequences and the second-generation high-throughput short sequences to produce a 729 Mb high-quality sequence assembly of this species. We built a chromosome-level assembly with 41 complete chromosomes using Hi-C technology. We also performed precise genome annotations, comparative genome analysis, and phylogenetic studies of Oligochaetes and other related species in Annelida using our high-quality genome assembly and the transcriptome data from multiple tissue samples.

## Materials and Methods

### Genome sequencing and assembly

One clitellata of *Metaphire vulgaris* (Chen, 1930) grown in Jiangsu, China was prepared for genome and transcriptome sequencing. It was about three month in age, with the length and width of 17 cm and 0.7 cm, respectively Genomic DNA was extracted from the head muscle of the earthworm using the QIAGEN^®^ Genomic DNA Extraction Kit (Cat#13323, Qiagen) according to the manufacturer’s instructions.

We sequenced the whole genome of *M. vulgaris* using PromethION single molecule platform (Oxford Nanopore) and Illumina NovaSeq sequencing platform. A DNA library was constructed following the standard Oxford Nanopore protocol and was sequenced on the PromethION platform (Oxford Nanopore Technologies, ONT, UK). A Paired-End (PE) library was simultaneously constructed according to the manufacturer’s instructions for genomic DNA sequencing (Illumina, San Diego, CA, USA). The insert size was approximately 400bp and was sequenced on an Illumina NovaSeq system (read length 150 bp).

Primers (5′-GGTCAACAAATCATAAAGATATTGG-3′ and 5′-TAAACTTCAGGGTG ACCAAAAAATCA-3′) were used to amplify the mitochondrial cytochrome c oxidase subunit I (COX1) gene from the extracted DNA. The PCR products were sequenced on the ABI 3730xl DNA Analyzer. And the sequences were compared by BLASTN (Altschul et al., 1990) with default settings.

We adopted a combined strategy of filtering, assembling, and polishing with multiple software pipelines to obtain a high-quality genome assembly of *M. vulgaris*. Canu version 1.8 (Koren et al., 2017) was used with default parameters to filter and correct the raw reads from the Nanopore high-noise single-molecule sequencing. SMARTdenovo (Istace et al., 2017) was used to assemble the contigs of the *M. vulgaris* genome (-c 1 -k 21). The ONT reads were re-aligned to the assembled contigs using racon v1.0.0 (default settings; https://github.com/lbcb-sci/racon) and minimap2 (v2.1, -x map-ont; Li, 2018) to reduce the assembly errors. The ONT contigs were polished three times by the PE reads, which were produced from whole-genome shotgun dataset using BWA (v0.7.17-r1188, default settings; Li & Durbin, 2009) and Pilon (v1.22, –changes –vcf –diploid –mindepth 10; Walker et al., 2014) to remove minor errors (SNP and indels). And BUSCO (Benchmarking Universal Single Copy Orthologs; Simão et al., 2015; v3.0.2, -m geno -l metazoa_odb9) was conducted to evaluate the assembled contigs by searching for 978 metazoa-conserved genes.

The Hi-C library was constructed following the method of Wang et al. (2015), with the DNA extracted from head muscle tissue of *M. vulgaris*. The 150 bp paired-end reads were sequenced using the Illumina NovaSeq system. Contigs were clustered and sorted into chromosomes by LACHESIS based on the Hi-C data (Burton et al., 2013; CLUSTER MIN RE SITES = 100; CLUSTER MAX LINK DENSITY = 2.5; CLUSTER NONINFORMATIVE RATIO = 1.4; ORDER MIN N RES IN TRUNK = 60; ORDER MIN N RES IN SHREDS = 60). Finally, placement and orientation errors exhibiting obvious discrete chromatin interaction patterns were manually adjusted. Briefly, a heat map was drawn by the bin matrix file from LACHESIS’s result. Then we retrieved the scatter points of the collinear signal that obviously did not conform to the correct positional relationship of the chromosomes, and revised the bin matrix file by adjusting the contig order or cutting off the contig to generate a new interactive heat map which was consistent with the chromosome spatial position signal. Purge Haplotigs (v1.1.1; Roach et al., 2018) was used to evaluate the genome assembly (default parameters), along with minimap2 (-ax map-ont) and SAMtools (v1.9, view -hF 256; Li et al., 2009). The Illumina paired-end data were mapped to assembled contigs using Bowtie2 (v2.2.6, -I 50 -X 1000; Langmead & Salzberg, 2012), and pile upped with SAMtools (mpileup -f).

### Karyological analysis and genome size estimation

One individual of *Metaphire vulgaris* (17 cm/0.7 cm in length/width, 3 months old) was prepared for the karyological analysis. 0.2 ml colchicine (1mg/ml) was injected into mature subjects of *M. vulgaris*. The testes and sperm sacs of the earthworm were isolated and triturated 24 h after the injection. The sample was dyed using 0.1ug/ml DAPI for 5–10 min and was observed under a fluorescence microscope (ZEISS Axio Imager2). Ikaros software (https://metasystems-international.com/cn/products/ikaros/) was used to analyze the karyotype.

The k-mer analysis software Kmerfreq_AR (SOAPec_v2.01 package https://sourceforge.net/projects/soapdenovo2/files/ErrorCorrection/SOAPec_v2.01.tar.gz/download) was adopted to estimate the genome size of *M. vulgaris* at k-mer 17.

### RNA preparation and sequencing

From the same individual used in genome sequencing, total RNA of six tissues (heart, ventral nerve cord, gonad, epidermis, intestine, and tail) was prepared. RNA degradation and contamination was monitored on 1% agarose gels. RNA concentration was measured using Qubit^®^ RNA Assay Kit in Qubit^®^ 2.0 Flurometer (Life Technologies,CA, USA). RNA integrity was assessed using the RNA Nano 6000 Assay Kit of the Bioanalyzer 2100 system (Agilent Technologies, CA, USA). A total amount of 1 µg RNA per sample was used as input material for the RNA sample preparations. Sequencing libraries were generated using TruSeq RNA Library Preparation Kit (Illumina, USA) following manufacturer’s recommendations and index codes were added to attribute sequences to each sample. The library preparations were sequenced on an Illumina Novaseq platform and 150 bp paired-end reads were generated. Fastp (version 0.12.6; Chen et al., 2018) with default parameters was applied to filter out low quality reads.

### Genome annotation

RepeatModeler (http://www.repeatmasker.org/RepeatModeler.html, version 1.0.5) was used to build the custom repeat library from the genome assembly sequence of *M. vulgaris*. The homologous repeat elements in the genome of *M. vulgaris* were identified and classified using RepeatMasker (http://www.repeatmasker.org/, version 3.3.0).

In order to build the preliminary gene models on the repeat-masked genome sequence. Augustus (Stanke et al., 2006; v3.2.1, –species=caenorhabditis) and SNAP (Korf, 2004; version 2006-07-28, C.elegans.hmm) were applied to predict *de novo* genes using gene model parameters trained by *Caenorhabditis elegans*. With the splice junctions identified by STAR (Dobin et al., 2013; STAR-2.6.1c, –outSAMtype BAM SortedByCoordinate –outFilterType BySJout –outFilterMultimapNmax 20 –alignSJoverhangMin 8 –alignSJDBoverhangMin 1 –outFilterMismatchNmax 999 –outFilterMismatchNoverLmax 0.04 –alignIntronMin 20 –alignIntronMax 20000 –alignMatesGapMax 20000 –chimSegmentMin 20), GeneMark-ET (Lomsadze, Burns & Borodovsky, 2014; v4.46, gmes_petap.pl –ET) was also used to perform unsupervised training with RNA-Seq data from six tissues (heart, ventral nerve cord, gonad, epidermis, intestine, and tail) and subsequently generates *ab initio* gene predictions. In the mean time, the filtered RNA-seq reads generated from six tissues were assembled through two approach: (1) RNA-seq reads were aligned to the genome assembly by HISAT2 (Kim et al., 2019; v2.0.5, –dta), and then imported to the genome-guided assembler StringTie (Pertea et al., 2015; v1.3.0, default settings); (2) RNA-seq reads were imported to the *de novo* assembler Trinity (Grabherr et al., 2011; v2.1.1, –normalize_reads –SS_lib_type FR). The two sets of assembled transcripts were reassembled based on the overlapping alignments by PASA (Campbell et al., 2006; v2.0.1, –ALIGNERS gmap -I 25000 -C -R). And the protein sequences of three close related species (*Capitella teleta*, *Helobdella robusta*, *Caenorhabditis elegans*, from EnsemblMetazoa (http://metazoa.ensembl.org/index.html) were also aligned to the genome assembly by Exonerate (Slater & Birney, 2005; v2.2.0, -m protein2genome –percent 50 –querytype protein –targettype dna) to find homologus genes. Finally, the predicted gene structures were integrated into consensus gene structures using EVidenceModeler (EVM; Haas et al., 2008; v1.1.1, –segmentSize 5000000 –overlapSize 100000, –weights PROTEIN 5; TRANSCRIPT gmap 5/assembler 10; ABINITIO_PREDICTION 5). Genes with expression evidences or protein homologues were regarded as high quality (HQ) genes.

The functional classification of Gene Ontology (GO; http://www.geneontology.org/) of the genes was performed by the InterProScan program (Zdobnov & Apweiler, 2001). RNA-seq reads generated from the six tissues were mapped to the coding sequence of genes by hisat2 v2-2.0.5 (Kim et al., 2019) using default parameters. Normalized read counts based on the gene annotation were calculated using R package DESeq2 (Love, Huber & Anders, 2014).

### Comparative genomics analysis

Protein-coding genes and CDS of *Caenorhabditis elegans*, *Lottia gigantea*, *Capitella teleta* and *Helobdella robusta* were downloaded from the EnsemblMetazoa database (https://metazoa.ensembl.org/info/data/ftp/index.html, release-45). Protein-coding genes and CDS of *Drosophila melanogaster*, *Danio rerio*, *Xenopus tropicalis*, *Gallus gallus*, *Homo sapiens*, and *Mus musculus* were downloaded from Ensembl database (http://www.ensembl.org/info/data/index.html, release-98). Only the longest transcript was selected for the genes with alternative splice variants. OrthoFinder (Emms & Kelly, 2015) was used to identify orthologs in these 12 species. The species evolution tree was constructed using Bayesian method based on single-copy gene families identified by OrthoFinder. The supergene sequences were subjected to phylogenetic analyses by mrbayes-3.2.7 (Ronquist et al., 2012) software with the parameter (mcmc ngen = 100000, samplefreq = 10), and *A. japonicus* was set as the outgroup. Then the first 25% samples from the cold chain (relburnin=yes and burninfrac = 0.25) were discarded. The Ks-based (Ks: synonymous substitution rate) ortholog age distributions were determined based on one-to-one orthologs between *M. vulgaris* and the other three species of Lophotrochozoa using default settings. The Ks estimation was calculated using KaKs_Calculator1.2 (Wang et al., 2009).

Intraspecific synteny analysis was performed for all gene models of *M. vulgaris* using MCscan (https://github.com/tanghaibao/jcvi/wiki/MCscan-(Python-version)) with default parameters. Ks of the paralog gene pairs in synteny blocks was also estimated by KaKs_Calculator1.2. Circos software (Krzywinski et al., 2009) was used for synteny visualization within the genome of *M. vulgaris*.

### Hox genes and lumbrokinase genes

Hox genes were identified using the homology search. The homeodomain sequences downloaded from the homeobox database (http://homeodb.zoo.ox.ac.uk/) were aligned using clustalw2 (Larkin et al., 2007). These sequences were used to construct a homeobox HMM using hmmbuild from the HMMER v3.1b suite (Eddy, 2011). Predicted proteins in the *M. vulgaris* genome were scanned using hmmsearch from HMMER v3.1b suite. The candidate genes belonging to the HOXL subclass of ANTP class were further filtered based on manual curation and molecular phylogeny. The subclass of these Hox genes was determined using the homeodomain regions of filtered Hox genes in *M. vulgaris*. The homeobox genes from spiralian genomes (Simakov et al., 2013) were used to construct the phylogenetic tree using the Neighbor-Joining method in MEGA7 (Kumar, Stecher & Tamura, 2016). Heat maps of Hox gene expression were drawn with the pheatmap package in R (https://cran.r-project.org/web/packages/pheatmap/index.html).

Amino acid sequences of 22 lumbrokinase gene families obtained from NCBI (AAL28118, AAN28692, AAN78282, AAP04532, AAP92795, AAR13225, AAR13226, AAR13227, AAT74899, AAT74900, AAW27919, ABA43718, ABQ23217, ABW04903, ABW04904, ABW04905, ABW04906, AIC77168, AKQ13274, ARD24433, ATP16189, QBA57435) were aligned to the gene models of *M. vulgaris* by BLASTP with e-value 1 × 10^−10^. Aligned hits with greater than 50% identity and 50% coverage were considered homologs of lumbrokinase, and the results were summarized manually.

## Results

### Genome sequencing and de novo assembly

The DNA fragment identified by PCR amplification and sequencing of the mitochondrial DNA (Zhang et al., 2016) was found to be nearly identical (99.7%) to the published *M. vulgaris* mitochondrial sequence, confirming the identity of *M. vulgaris*. Karyological analysis allowed us to verify as many as 41 pairs of chromosomes for *M. vulgaris*. One chromosome in particular was shown to be much larger than the other chromosomes (Fig. S1).

Approximately 37 gigabase (Gb) single molecule reads (over 50-fold genome coverage) and 45 Gb short reads were generated and used to assemble the whole genome of *M. vulgaris*. The estimated genome size of *M. vulgaris* was determined to be about 0.65 Gb based on analysis of 17-mer sequences on short reads. The *k*-mer distribution showed that the genome is highly heterozygous (Fig. S2). A high-quality genome assembly of *M. vulgaris* was obtained using the combined strategy of filtering, assembling, and polishing with multiple software pipelines. A total of 559 contigs were generated with a total length of 729 Mb and an N50 size of 4.2 Mb (Table 1). The largest contig reached the length of 16.6 Mb. Chromosome conformation capture (Hi-C) and short read sequencing were used to assemble the contigs into chromosomes, resulting in 104 Gb raw data (∼150-fold).

**Table 1:** *Metaphire vulgaris* genome statistics and gene predictions.

**Assembly feature**	
Assembled sequences	728,570,957 bp
N50 contig length	4,202,844 bp
N90 contig length	649,861 bp
Longest contig	16,632,089 bp
Number of contigs	559
N50 scaffold length	16,345,198 bp
N90 scaffold length	12,220,767 bp
Longest scaffold	50,191,389 bp
Number of scaffold	280
** Repeat sequences**	
SINEs	2,444,842 bp (0.34%)
LINEs	73,457,768 bp (10.8%)
LTR elements	8,821,633 bp (1.21%)
DNA elements	59,603,311 bp (8.18%)
Total repeats	334,237,560 bp (45.88%)
**Gene annotation**	
Gene models (high confidence)	43,842
Gene models (low confidence)	6,397
Average gene length	6,154.5 bp
Average CDS length	1,185.3 bp

**Notes.**

a The average gene and CDS length were estimated based on the gene models with high confidence (HQ gene models).

The assembled *M. vulgaris* contigs were clustered and sorted according to the Hi-C data, with the assembly errors corrected. We obtained 41 major groups corresponding to 41 chromosomes (MV001∼MV041, Fig. S3), representing 95.71% of all the contigs. The largest chromosome was as long as 50.2 Mb, with the others ranging from 11.0 to 30.9 Mb (Table S1), which was consistent with the karyological analysis. Purge Haplotigs showed that the estimated haplotigs and artefacts were only 24,929,850 bp (3.42% of the whole assembly 728,570,957 bp) and 442,973 bp (0.06%), respectively. And the haplotigs and artefacts were all belonged to the small and unlocated scaffolds other than the 41 chromosomes. Read coverage statistics indicated that approximately 93.5% of raw reads could be aligned with the final assembly, covering 99.6% of the genome. The BUSCO results indicated that 94.3% (922) of genes were completely captured (81.2%/794 were complete and single-copy BUSCOs, 13.1%/128 were complete and duplicated BUSCOs), 1.2% (12) were fragmented, and only 4.5% (44) were missing from the assembly.

### Genome annotation

The *M. vulgaris*-specific repetitive sequences were *de novo* identified, which was followed by the whole genome screening. The overall repeat content of *M. vulgaris* was approximately 45.88%, of which approximately 22.0% and 17.8% were LINEs and DNA elements, respectively. The proportion of repetitive elements in *M. vulgaris* was much greater than that was indicated in a previous study of *Hirudinaria manillensis* (19.52%) and *Helobdella robusta* (17.33%) (Guan et al., 2020), the two species of leech also belonging to the Annelida. The contrast in repetitive element content may be the reason for the larger genome size of *M. vulgaris*.

**Figure 1:**
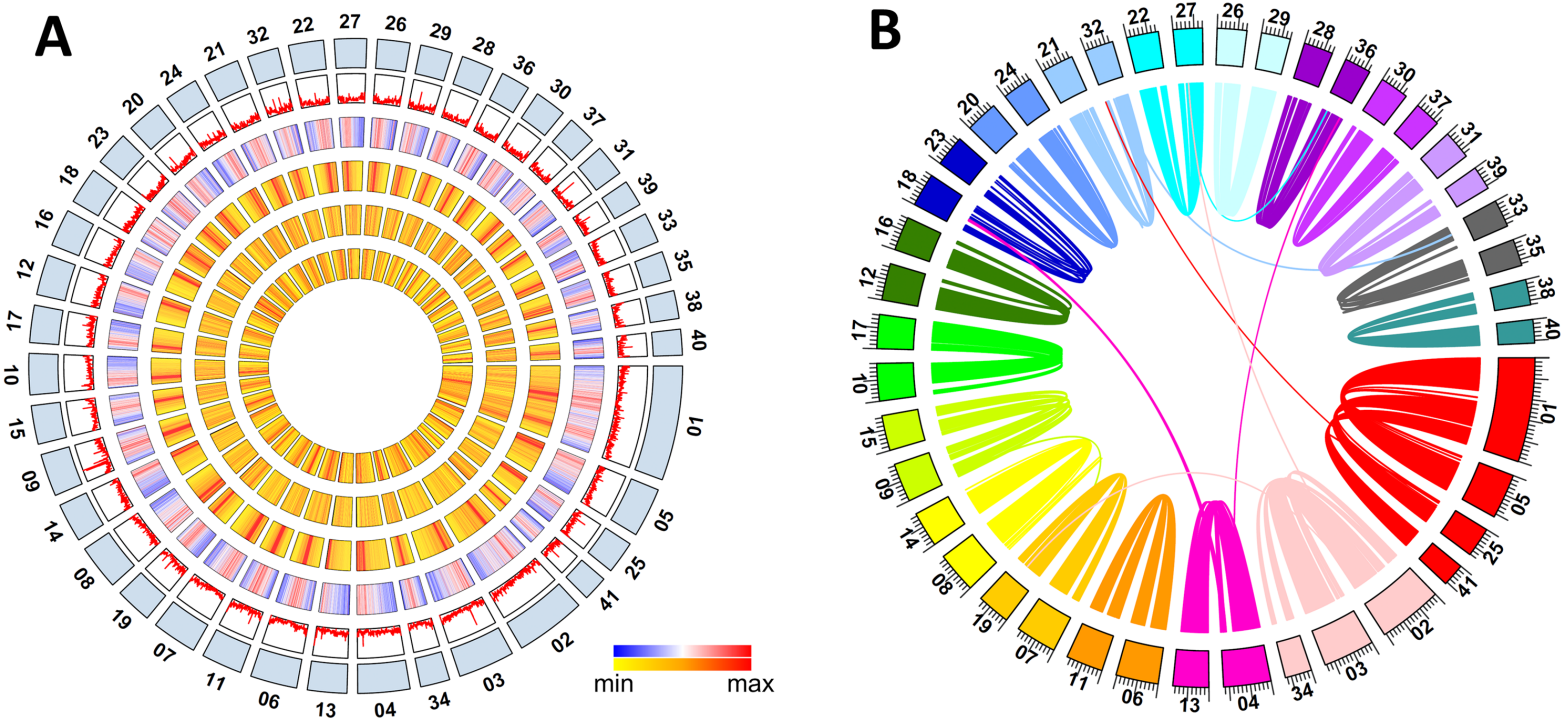
*Metaphire vulgaris* genome features. (A) Characteristics of the 41 chromosomes of *M. vulgaris*. From the outer to the inner circles are: The chromosomes; gene density; density of DNA elements; density of LINE; density of SINE; density of LTR (densities shown as percent nucleotides per 100 Kb). (B) Syntenic blocks within the *M. vulgaris* genome (6,453 gene pairs, 298 blocks which consisted of four continuous genes at least).

A combined strategy of ab initio predictions, RNA-seq supported evidence, and homologous searching was used to obtain a reliable protein-coding gene set. RNA-seq reads with an average of 8.6 Gb raw data per tissue were generated from six tissues (heart, ventral nerve cord, gonad, epidermis, intestine, and tail) and used to generate a set of high quality (HQ) gene models. 50,239 gene models were created from the EvidenceModeler (EVM) pipeline; 43,842 of them were HQ genes with expression evidences or protein homologues, which were also assessed by BUSCO (Table S2). The average gene length was 6,154.5 bp among the HQ genes, the average coding sequence (CDS) length was 1,185.3 bp, and the average exons per gene was 5.7. The gene length of *M. vulgaris* was much larger than the Asian buffalo leech but the CDS lengths were similar (Guan et al., 2020) (Table S3). The genome features, including the distribution of genes and repeats along the chromosomes, are shown in Fig. 1A.

### Evolutionary and comparative genomic analysis

Intraspecific synteny analysis was performed with all the gene models of *M. vulgaris* using self pair-to-pair alignment. Approximately 25.7% of the protein coding genes had synteny blocks (6,453 gene pairs, 298 blocks with at least four continuous genes) within the assembled genome. Intra-genomic gene comparison showed that co-linearity was distributed along almost the entire 41 chromosomes (Fig. S4), indicating the whole genome duplication occurred during the evolution of the *M. vulgaris* genome (Fig. 1B). Chromosome fusion events could also be inferred, including the event in which three chromosomes merged into chromosome number one (MV001), and two chromosomes merged into chromosome number two (MV002), creating the unusually large size of the two largest chromosomes (50.2 Mb and 30.9Mb, respectively). Gene synteny analysis between *M. vulgaris* and other species (*H. robusta*, *C. teleta*, *L. gigantea*, *C. elegans*) was also conducted. However, few instances of gene synteny could be identified in all genomes, revealing the different genome structure and genome evolution of *M. vulgaris*.

**Figure 2:**
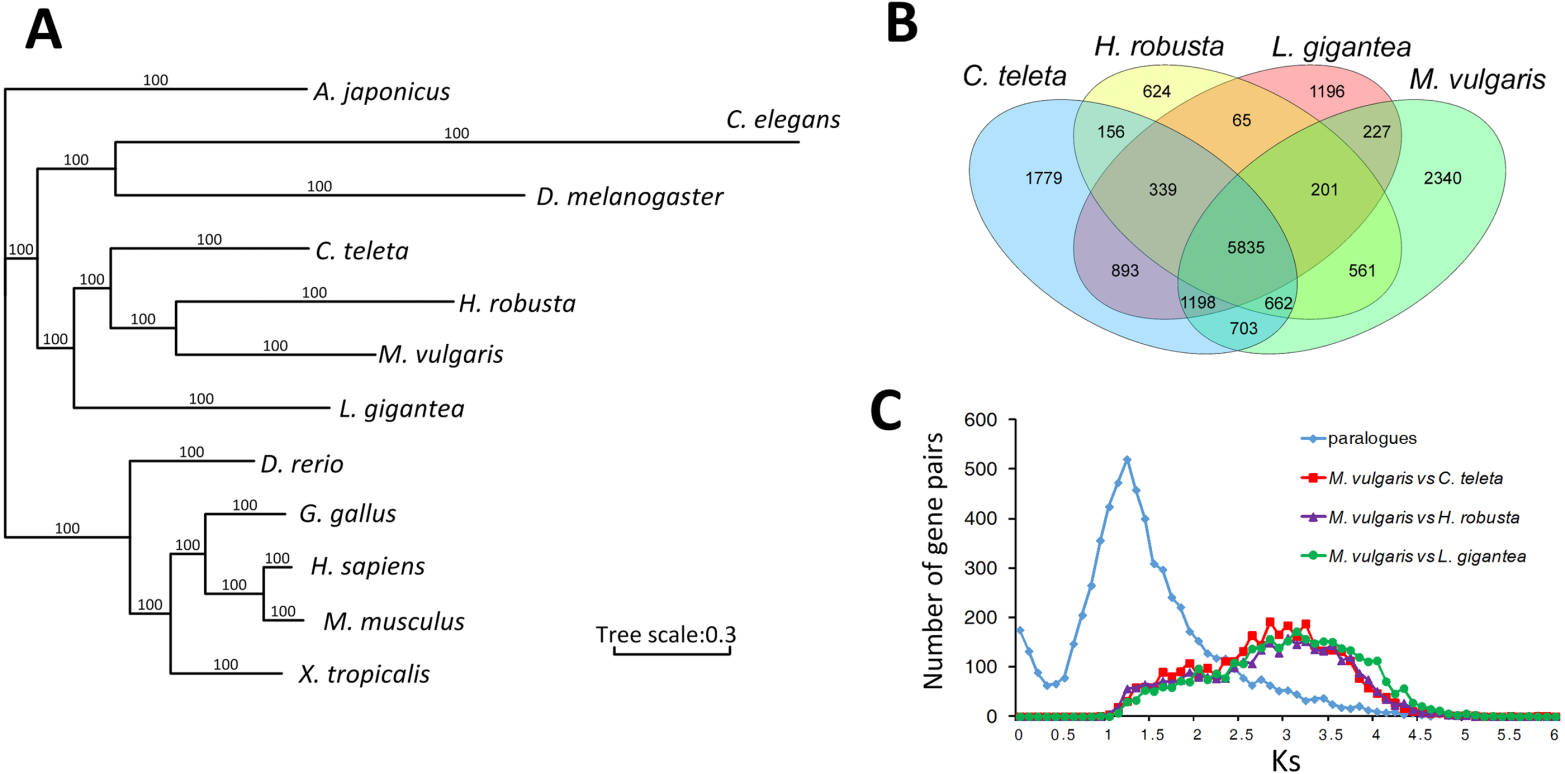
Comparison of homologue genes between *M. vulagris* and other species. (A) Phylogenic tree shows the relationships among metazoans by whole genome evidence of ortholog single copy gene families (*A. japonicus* was set as the outgroup); (B) Venn plot of the common identified gene families among the four Lophotrochozoa Genomes; (C) the Ks distributions of ortholog genes between *M.vulgaris* and other three Lophotrochozoa species, along with the Ks of paralog genes within *M.vulgaris*.

A total of 310 singlecopy ortholog gene families were selected from *M. vulgaris* and eleven other published animals, including *Drosophila melanogaster*, *Caenorhabditis elegans*, *Lottia gigantea*, *Capitella teleta*, *Helobdella robusta*, *Apostichopus japonicus*, *Danio rerio*, *Xenopus tropicalis*, *Gallus gallus*, *Homo sapiens*, and *Mus musculus*. *M. vulgaris* and *H. robusta* (leech) are in the same branch of the phylogenetic tree with *C. teleta* in the nearest branch (Fig. 2A). All three species are Annelida but *C. teleta* is considered to be more primordial than the other two (Simakov et al., 2013), which is supported by the whole genome data. Common gene families were identified from the four Lophotrochozoa genomes (Fig. 2B), which showed that *M. vulgaris* had 2,340 specific genes. A one-to-one comparison of the ortholog genes of *M. vulgaris* and the other three Lophotrochozoa species determined that the divergence of *M. vulgaris* was similar, while the Ks distribution of paralog genes within *M. vulgaris* showed a much earlier peak (Fig. 2C).

### Hox genes and lumbrokinase genes

**Figure 3:**
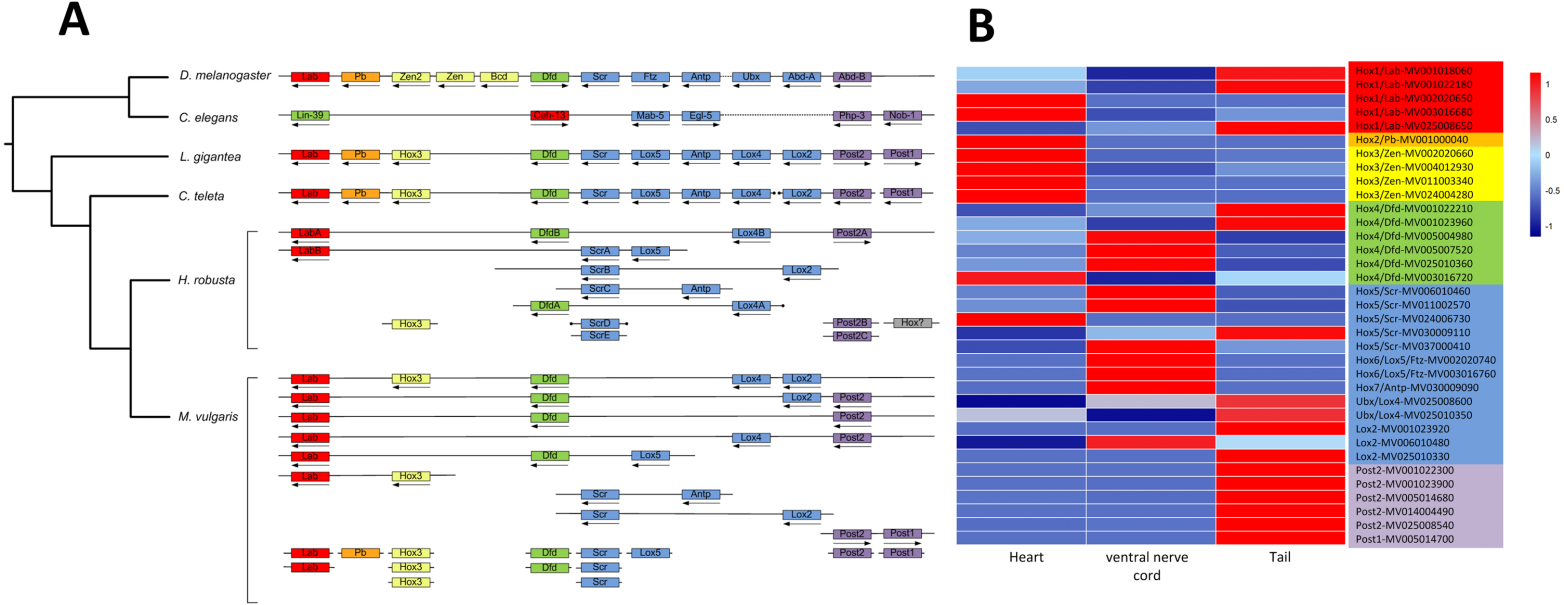
Hox gene complex and their expression in *M. vulgaris*. (A) The clusters of Hox genes in *M. vulgaris* and other five species. All the six species have been sequenced and assembly with good quality. The arrows, dots, and colors were defined as those from Simakov et al. (2013). Red, orange, yellow, green, blue, purple were assigned to paralog groups (Hox1, Hox2, Hox3, Hox4, central class and posterior class). Arrows indicated the direction of transcription. The ends of the scaffolds were marked by black dots; (B) the expression of the Hox genes in heart, ventral nerve cord and tail of *M. vulgaris*. The colors of the gene name on the right side were according to the Hox paralog groups described above.

A total of 343 homeobox genes were retrieved from *M. vulgaris*, which is consistent with previous studies (Zwarycz et al., 2015). 41 Hox genes, which belong to the HOXL subclass of ANTP class, were identified by a homeodomain search (Fig. S5). Among them, 28 genes were clustered in 9 chromosomes. The Hox gene clusters of *M. vulgaris* and their paralog groups in other five genomes (*D. melanogaster*, *C. elegans*, *L. gigantean*, *C. teleta*, *H. robusta*) were analyzed together due to their relatively good genome assemblies. There were more Hox gene clusters in *M. vulgaris* than in other species but the order of the paralog genes was similar (Fig. 3A, Table S4) and the distribution pattern was similar to that of *H. robusta*, which is also in Annelida. The expression levels of the Hox genes in *M. vulgaris* were evaluated in the heart, ventral nerve cord, and tail indicating that *Hox1(Lab)/Hox2(Pb)/Hox3(Zen)* were mostly expressed in the heart, *Hox4(Dfd)/Hox5(Scr)/Hox6(Lox5)/Hox7(Antp)/Ubx(Lox4)/Lox2* were mostly expressed in the ventral nerve cord, and *Post2/Post1* were expressed in the tail (Fig. 3B). This showed tissue-specific expression within the genes of the Hox paralog groups.

By homologue search against the *M. vulgaris* genes, twenty lumbrokinase-like genes were found within the whole genome and most of them were tandemly arranged (Fig. S6, Table S5). They were distributed on six chromosomes (MV001, MV005, MV010, MV017, MV020 and MV026) and one unanchored contig (MV154).

## Discussion

The karyotype information for earthworms in Lumbricidae was published many years ago (Gregory & Hebert, 2002), revealing that the C-values ranged from 0.43–1.20 and chromosome numbers 2*n* = 22–190. However, there have been no recent studies on the chromosomes of any species in Megascolecidae and there has been no whole genome sequencing for the species in the *Metaphire* genus, with the exception of the mitochondrial genome (Zhang et al., 2016). We performed karyological analysis of this earthworm to verify the number of its chromosomes to build a better genome assembly of the newly sequenced species, *M. vulgaris*. 41 pairs of chromosomes were identified, which corresponded to the number of clusters obtained from Hi-C data. Intraspecific synteny analysis showed a whole genome duplication of the *M. vulgaris*, followed by several chromosome fusion events, revealing the evolutionary route from an ancient tetraploid to a modern diploid species. The genome duplication (tetraploidization) may have led to the bigger body size of *M. vulgaris* versus other common earthworms.

Previous studies reported on two genome assemblies of earthworms (Zwarycz et al., 2015; Bhambri et al., 2018), both on *Eisenia fetida*, from the Lumbricidae family. Neither study produced a high-quality chromosome level genome assembly. The N50 scaffold lengths were only 9.31 Kb and 1.85 Kb, respectively, which was not long enough to identify an intact gene model. We generated high-quality genome and transcriptome datasets for *M. vulgaris* and created the first chromosome-level genome assembly of the oligochaete species, with almost all of the contigs clustered into 41 groups or chromosomes. The N50 contig length of the *M. vulgaris* genome assembly was as long as 4.2 Mb, which was hundreds of times larger than those of *Eisenia fetida*. This assembly was suitable for gene modeling and synteny analysis. The subsequent results of gene annotation, gene expression, and genome structure will provide valuable information for gene function and evolutionary studies.

Hox genes are a subset of homeobox genes and are a type of animal gene that specifically regulates biological structures. The Hox genes tend to be clustered in the genome and show a collinear correspondence between gene order and the body levels where these genes are expressed during development (Duboule, 2007). In our findings, Hox genes partial clustered around the *M. vulgaris* genome and showed tissue-specific expression. Lumbrokinase is a six-enzyme protein with fibrinolytic activity, which was first isolated from the crude of *Lumbricus rubellus* (Hoffmeister, 1843; Mihara et al., 1991). Fibrinolytic enzymes have been purified and characterized in *Eisenia foetida*, (Hrzenjak et al., 1998; Li, Zhao & He, 2003; Wang et al., 2003). And lumbrokinase is a medicinally valuable enzyme used to dissolve thrombi, reduce blood viscosity, and inhibit platelet aggregation (Wang et al., 2013). The identification of lumbrokinase-like genes of *M. vulgaris* were performed in the whole genome level, which indicated that they might tend to be clustered or tandemly distributed in earthworm genomes. The high-quality genome assembly and annotation of *M. vulgaris* will lay the foundation for the study of functional genes in earthworms, such as lumbrokinase and drilodefensin (Liebeke et al., 2015).

## Conclusions

We sequenced, assembled, annotated, and analyzed the genome of the *M. vulgaris*, which belongs to the genus *Metaphire* of the family Megascolecidae. The assembled sequence consisted of 559 contigs, with a length of 729 Mb, and an N50 contig size of 4.2 Mb. The contigs were anchored onto 41 chromosomes according to the Hi-C result, which was verified by karyological analysis. Whole-genome duplication and chromosome fusion events have been observed within the *M. vulgaris* genome. Hox genes and lumbrokinase genes were identified at the whole genome level and both of them were distributed as partial clusters. The high-quality genome assembly of *M. vulgaris* may be a valuable resource for genetic studies, gene cloning, and phylogenetic analysis in earthworms.

##  Supplemental Information

10.7717/peerj.10313/supp-1 Supplemental Information 1 Karyological analysis of *Metaphire vulgaris* showing that there were 41 pairs of chromsomes per cell The red arrow indicated the largest choromosome. (A) Haploid cell (*n* = 41); (B) Diploid cell (*n* = 82).

10.7717/peerj.10313/supp-2 Supplemental Information 2 K-mer distribution of *Metaphire vulgaris* short reads (*k* = 17) K-mer values were plotted against the frequency (*y*-axis) at their occurrence (*x*-axis). The estimated genome size of *M. vulgaris* is about 650 Mb. The left peak showed the high heterozygosity of the genome.

10.7717/peerj.10313/supp-3 Supplemental Information 3 Hi-C map of *Metaphire vulgaris* chromosomes showing genome wide chromatin interactions

10.7717/peerj.10313/supp-4 Supplemental Information 4 Intra-genomic comparison within *M. vulgaris* by 6,453 gene pairs

10.7717/peerj.10313/supp-5 Supplemental Information 5 Forty one Hox genes identified in *M. vulgaris* genome, along with the Hox genes from spiralian genomes Red, orange, yellow, green, blue, purple were assigned to paralog groups (Hox1, Hox2, Hox3, Hox4, central class and posterior class). MV003016720 in green belonged to Hox4, which was based on the BLASTP hits to NR (Non-Redundant Protein Sequence Database).

10.7717/peerj.10313/supp-6 Supplemental Information 6 Distribution of 20 Lumbrokinase genes on *M. vulgaris* chromosomes Tandem genes were labeled as purple triangles.

10.7717/peerj.10313/supp-7 Table S1 Characterization of the 41 *M. vulgaris* pseudo-chromosomes

10.7717/peerj.10313/supp-8 Table S2 Completeness of the assembled contigs and the high-confidence (HC) gene annotation as assessed by BUSCO

10.7717/peerj.10313/supp-9 Table S3 Statistics of the genes within three species in Annelida

10.7717/peerj.10313/supp-10 Table S4 Characterization of Hox genes in M. vulgaris

10.7717/peerj.10313/supp-11 Table S5 Characterization of the Lumbrokinases in *M. vulgaris*

10.7717/peerj.10313/supp-12 Supplemental Information 7 Gff3 file for all the *M. vulgaris* gene models

10.7717/peerj.10313/supp-13 Supplemental Information 8 Coding sequences of the *M. vulgaris* gene models

10.7717/peerj.10313/supp-14 Supplemental Information 9 Amino acid sequences of the *M. vulgaris* gene models

10.7717/peerj.10313/supp-15 Supplemental Information 10 Genome assembly of *M. vulgaris*, part1 (MV001∼MV003)

10.7717/peerj.10313/supp-16 Supplemental Information 11 Genome assembly of *M. vulgaris*, part2 (MV004∼MV008)

10.7717/peerj.10313/supp-17 Supplemental Information 12 Genome assembly of *M. vulgaris*, part3 (MV009∼MV014)

10.7717/peerj.10313/supp-18 Supplemental Information 13 Genome assembly of *M. vulgaris*, part4 (MV015∼MV020)

10.7717/peerj.10313/supp-19 Supplemental Information 14 Genome assembly of *M. vulgaris*, part5 (MV021∼MV027)

10.7717/peerj.10313/supp-20 Supplemental Information 15 Genome assembly of *M. vulgaris*, part6 (MV028∼MV035)

10.7717/peerj.10313/supp-21 Supplemental Information 16 Genome assembly of *M. vulgaris*, part7 (MV036∼MV280)

## Additional Information and Declarations

### Field Study Permissions

The following information was supplied relating to field study approvals (i.e., approving body and any reference numbers): The earthworms were collected in a typical farmland habitat with the permission of the farmer Genqing Wang from Yilong Farm (Jiangsu, China).

### Data Availability

The following information was supplied regarding data availability: Raw-data and the genome assembly are available at GenBank: PRJNA656665. This includes the Illumina whole-genome shotgun sequence (SRR12458316), Oxford Nanopore sequence (SRR12436614) and Hi-C raw-data (SRR12458315) of *M. vulgaris*; and the RNA-seq data for the six tissues (SRR12458313, SRR12458314, SRR12458317–SRR12458320). Raw data are also available at Bioproject: PRJCA002730.
