# Supplementary material for: High-quality genome assembly of Metaphire vulgaris
Source: PeerJ. 2020 Nov 12;8:e10313. doi: 10.7717/peerj.10313 (PMC7666815; doi:10.7717/peerj.10313)
Supplement: Supplemental Information 1 — The red arrow indicated the largest choromosome. (A) Haploid cell (n = 41); (B) Diploid cell (n = 82). [file peerj-08-10313-s001.pdf]

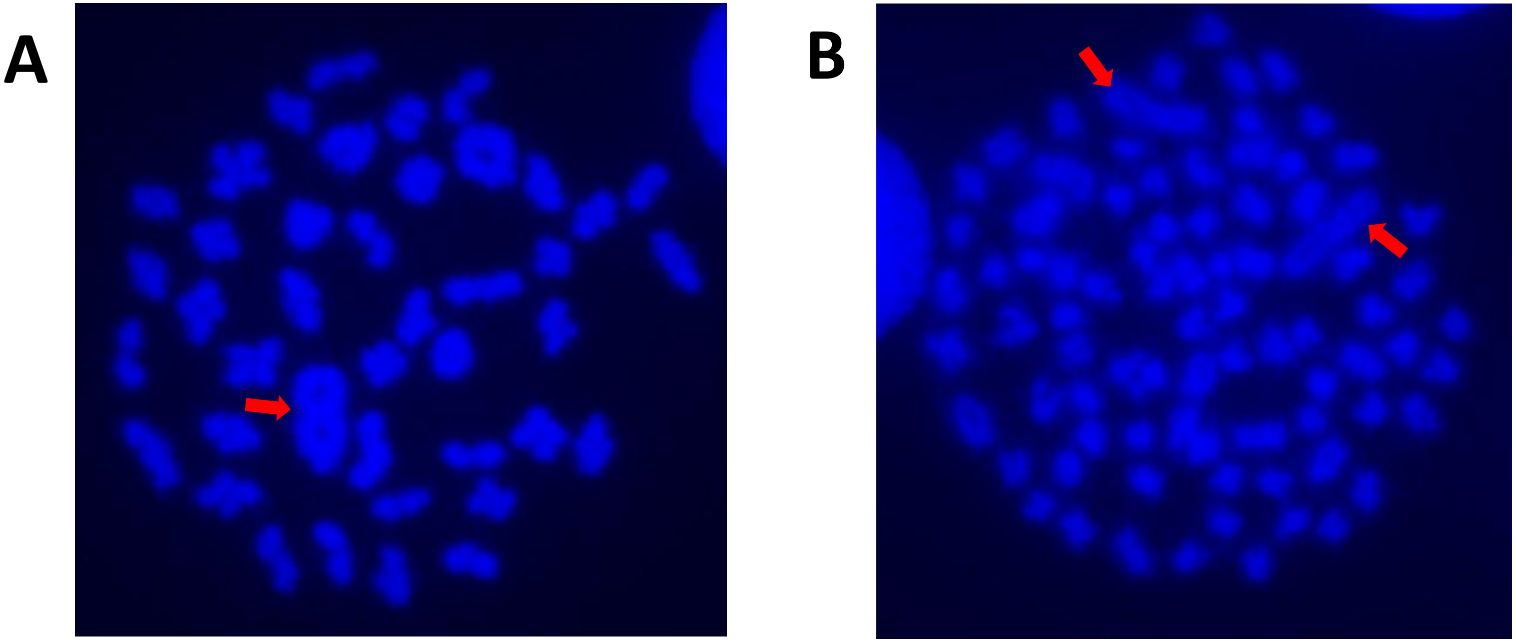

Figure S1. Karyological analysis of *Metaphire vulgaris* showing that there were 41 pairs of chromsomes per cell. The red arrow indicated the largest choromosome. (A) Haploid cell (n=41); (B) Diploid cell (2n=82).
