# Supplementary material for: High-quality genome assembly of Metaphire vulgaris
Source: PeerJ. 2020 Nov 12;8:e10313. doi: 10.7717/peerj.10313 (PMC7666815; doi:10.7717/peerj.10313)
Supplement: Supplemental Information 2 — K-mer values were plotted against the frequency (y-axis) at their occurrence (x-axis). The estimated genome size of M. vulgaris is about 650 Mb. The left peak showed the high heterozygosity of the genome. [file peerj-08-10313-s002.pdf]

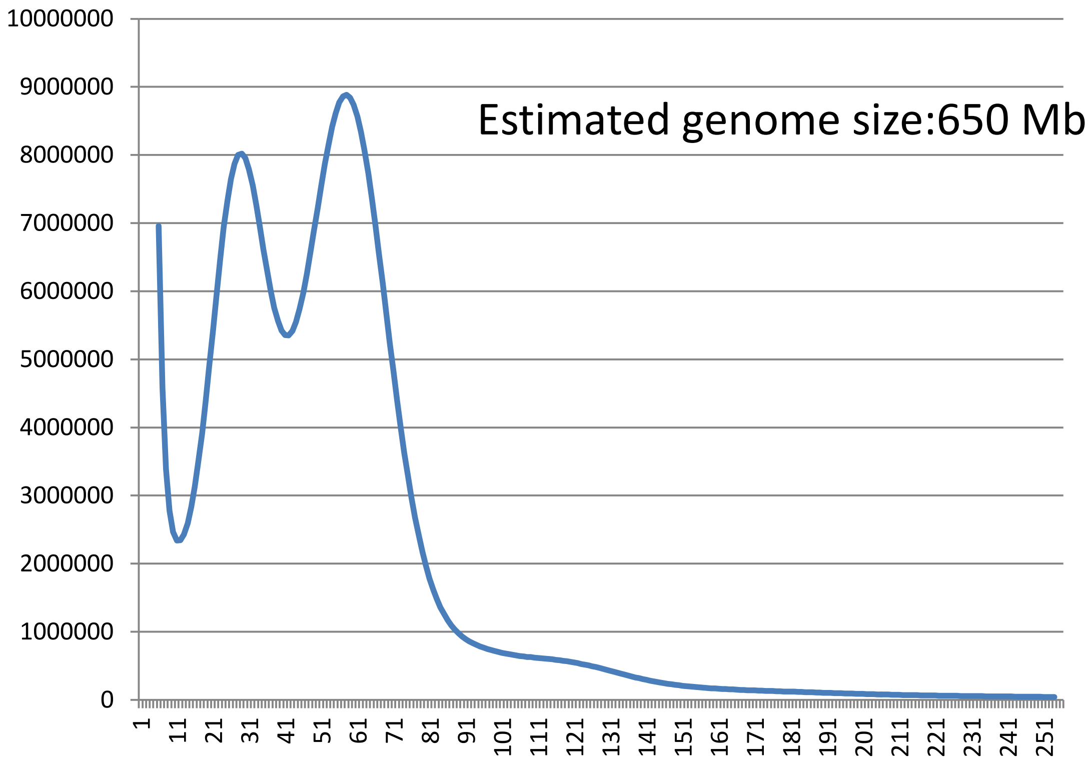

Figure S2. K-mer distribution of *Metaphire vulgaris* short reads (k=17). K-mer values were plotted against the frequency (y-axis) at their occurrence (x-axis). The estimated genome size of *M. vulgaris* is about 650 Mb. The left peak showed the high heterozygosity of the genome.
