# Supplementary material for: High-quality genome assembly of Metaphire vulgaris
Source: PeerJ. 2020 Nov 12;8:e10313. doi: 10.7717/peerj.10313 (PMC7666815; doi:10.7717/peerj.10313)
Supplement: Supplemental Information 3 [file peerj-08-10313-s003.pdf]

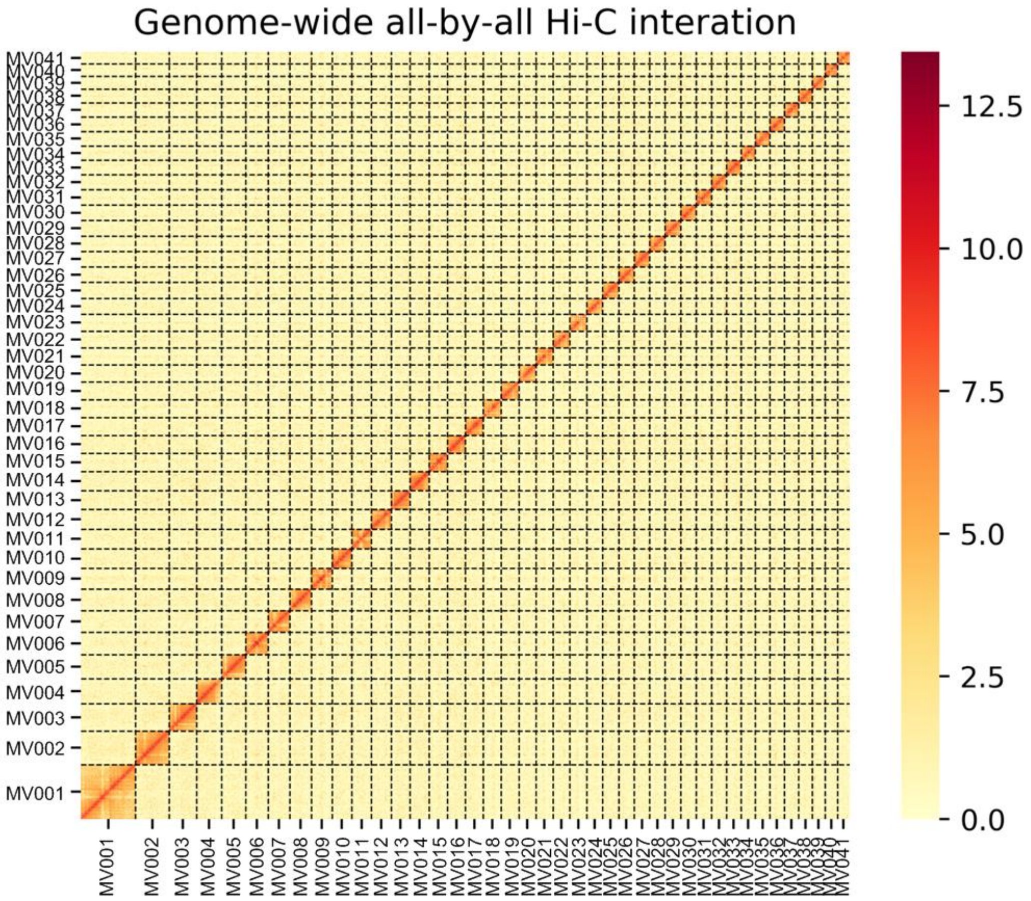

Figure S3. Hi-C map of *Metaphire vulgaris* chromosomes showing genome wide chromatin interactions.
