# Supplementary figures and images for: High-quality genome assembly of Metaphire vulgaris
Source: PeerJ. 2020 Nov 12;8:e10313. doi: 10.7717/peerj.10313 (PMC7666815; doi:10.7717/peerj.10313)

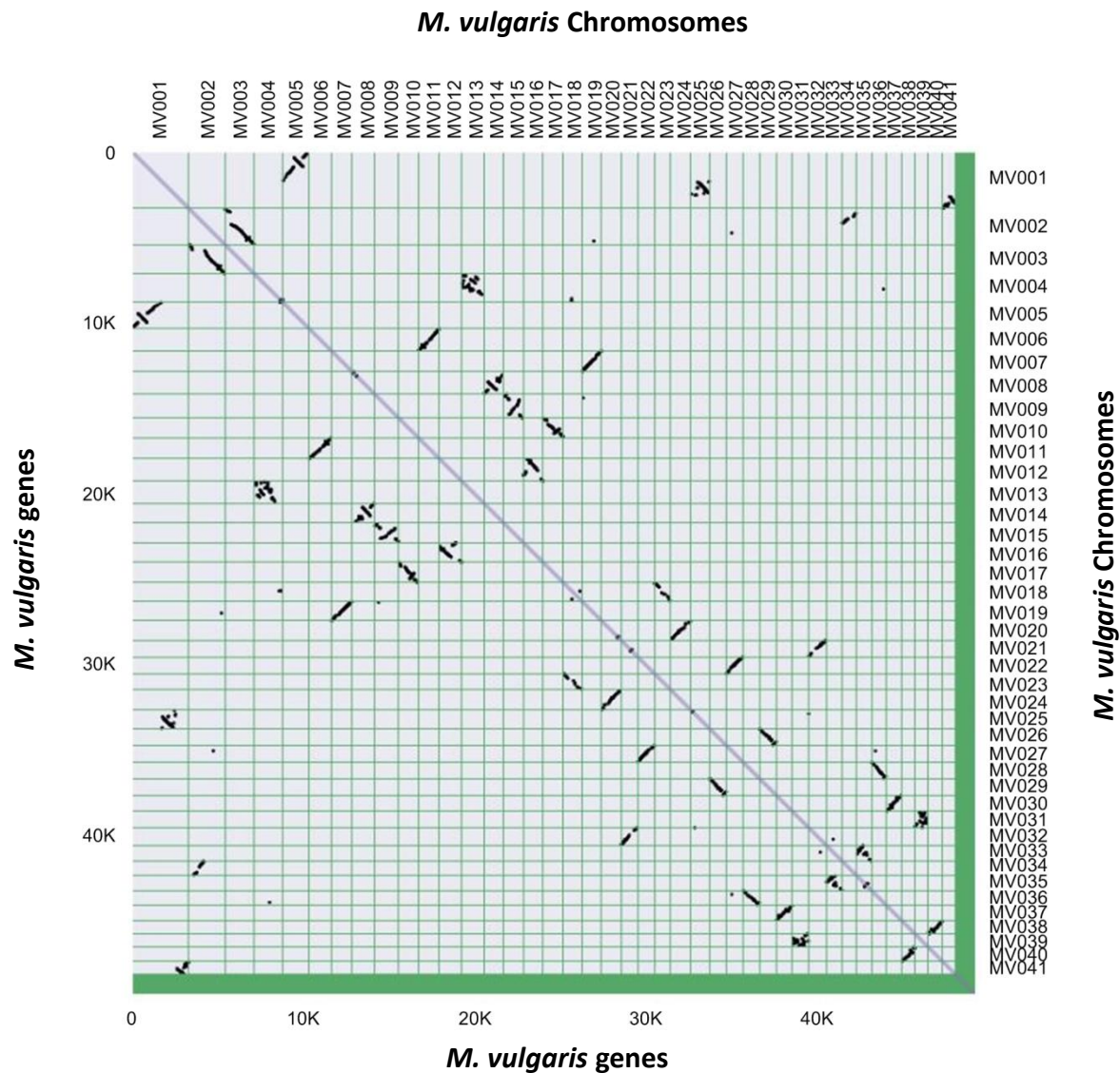

**Figure S4. Intra-genomic comparison within *M. vulgaris* by 6,453 gene pairs.**

Supplement: Supplemental Information 4 [file peerj-08-10313-s004.pdf]
