# Supplementary material for: High-quality genome assembly of Metaphire vulgaris
Source: PeerJ. 2020 Nov 12;8:e10313. doi: 10.7717/peerj.10313 (PMC7666815; doi:10.7717/peerj.10313)
Supplement: Supplemental Information 5 — Red, orange, yellow, green, blue, purple were assigned to paralog groups (Hox1, Hox2, Hox3, Hox4, central class and posterior class). MV003016720 in green belonged to Hox4, which was based on the BLASTP hits to NR (Non-Redundant Protein Sequence Database). [file peerj-08-10313-s005.pdf]

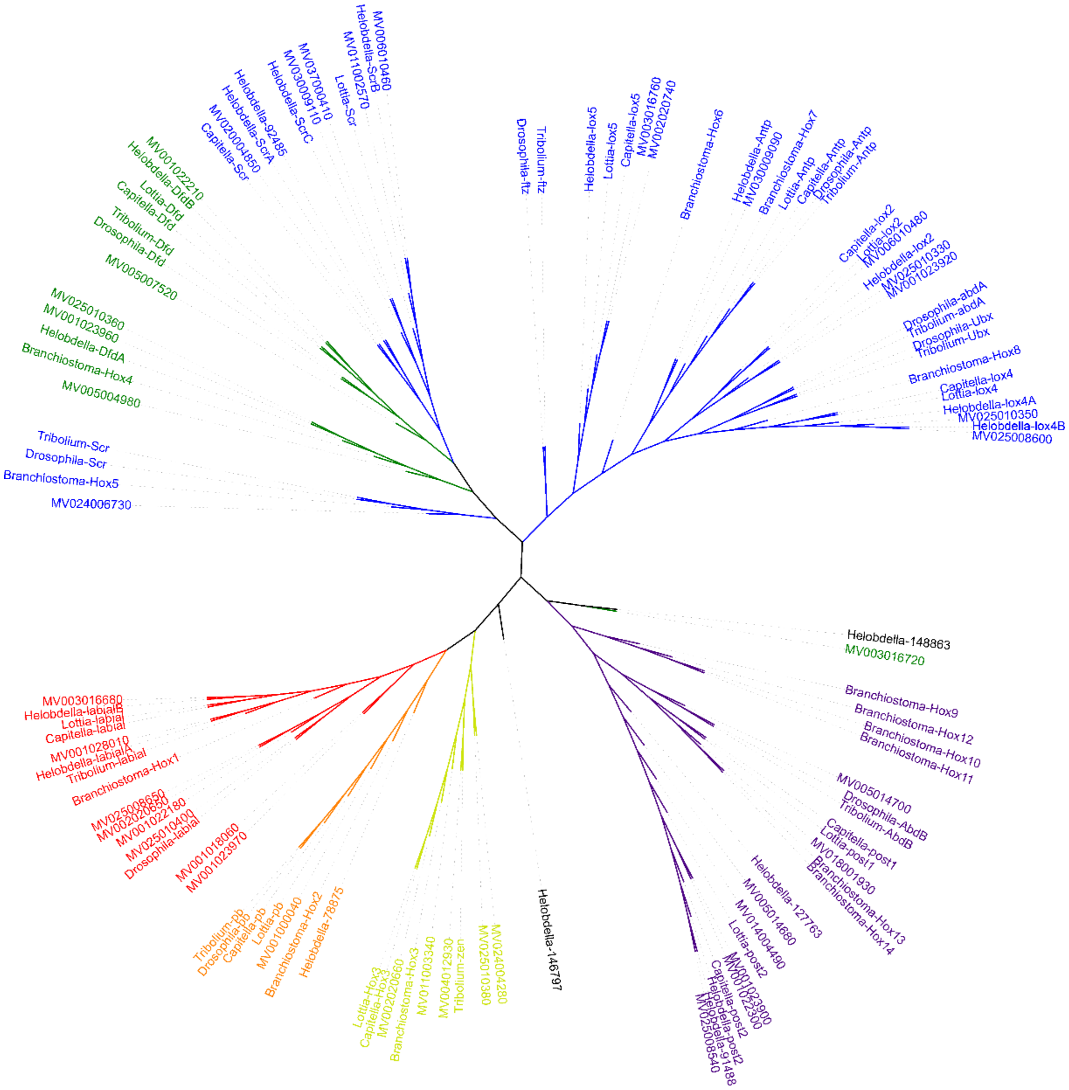

Figure S5. Forty one Hox genes identified in *M. vulgaris* genome, along with the Hox genes from spiralian genomes. Red, orange, yellow, green, blue, purple were assigned to paralog groups (Hox1, Hox2, Hox3, Hox4, central class and posterior class). MV003016720 in green belonged to Hox4, which was based on the BLASTP hits to NR (Non-Redundant Protein Sequence Database).
