# Supplementary material for: High-quality genome assembly of Metaphire vulgaris
Source: PeerJ. 2020 Nov 12;8:e10313. doi: 10.7717/peerj.10313 (PMC7666815; doi:10.7717/peerj.10313)
Supplement: Supplemental Information 6 — Tandem genes were labeled as purple triangles. [file peerj-08-10313-s006.pdf]

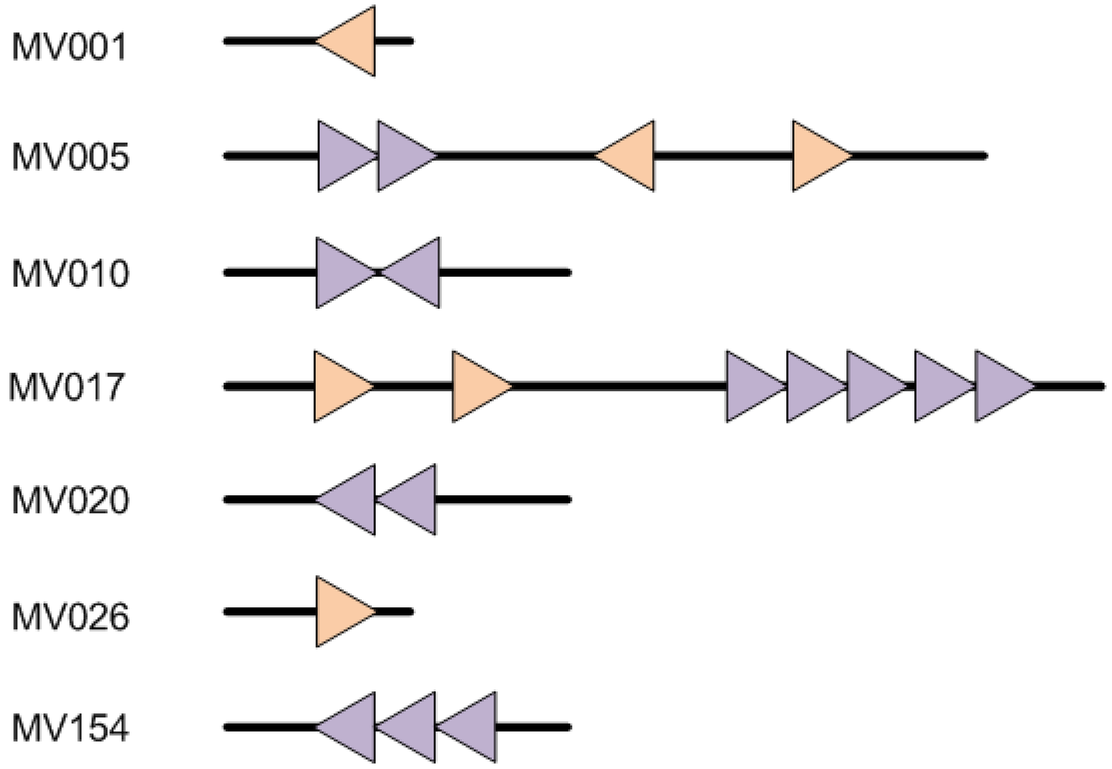

Fig. S6. Distribution of 20 Lumbrokinase genes on *M. vulgaris* chromosomes.
Tandem genes were labeled as purple triangles.
